# Novel method to analyze cell kinetics for the rapid diagnosis and determination of the causative agent in allergy

**DOI:** 10.1371/journal.pone.0246125

**Published:** 2021-02-19

**Authors:** Hirotomo Shibaguchi, Yuki Yasutaka, Koujiro Futagami

**Affiliations:** 1 Department of Biochemistry, Faculty of Medicine, Fukuoka University, Fukuoka, Japan; 2 Department of Hospital Pharmacy, Fukuoka University Hospital, Fukuoka, Japan; 3 Department of Health Care Management, Faculty of Pharmaceutical Science, Fukuoka University, Fukuoka, Japan; MAHSA University, Malaysia, MALAYSIA

## Abstract

Drug-induced allergy (DIA), an unexpectedly triggered side effect of drugs used for therapeutic purposes, is a serious clinical issue that needs to be resolved because it interrupts the treatment of the primary disease. Since conventional allergy testing is insufficient to accurately predict the occurrence of DIA or to determine the drugs causing it, the development of diagnostic and predictive tools for allergic reactions is important. We demonstrated a novel method, termed high-sensitive allergy test (HiSAT), for the rapid diagnosis of allergy (within 1 hr; with true-positive diagnosis rates of 89% and 9% for patients with and without allergy-like symptoms, respectively). HiSAT analyzes the cell kinetics as an index against chemotactic factors in a patient’s serum, as different from the diagnosis using conventional methods. Once allergy has occurred, HiSAT can be used to determine the causative medicine using culture supernatants incubated with the subject’s lymphocytes and the test allergen. This test is more efficient (60%) than the lymphocyte transformation test (20%). Furthermore, in HiSAT, cell mobility significantly increases in a dose-dependent manner against supernatant incubated with lymphocytes from a subject with pollinosis collected at a time when the subject is without allergic symptoms and the antigen. The result demonstraed that HiSAT might be a promising method to rapidly diagnose DIA or to determine with high accuracy the antigen causing allergy.

## Introduction

Drug-induced allergy (DIA) is triggered when drug molecules, their metabolites, or the protein–drug complexes, which are directly recognized as allergens, induce allergic inflammation [1, 2]. Humoral factors including cytokines and/or chemokines, which are essential for allergic inflammation, are released and promote the migration of immune cells (such as eosinophils, macrophages, and T cells) toward the local sites of allergic reactions [3–5]. Allergic diseases are complex disorders associated with different cellular and molecular mechanisms (such as signaling cascades of cytokines/chemokines, differentiation or proliferation of immune cells) [6–9]. Once DIA occurs in a patient, the drugs used for therapy are discontinued and the patient is treated for the allergic symptoms. Therefore, it is important to develop methods for accurate diagnosis and correct treatment in allergy.

Although the lymphocyte transformation test (LTT; also called the drug-induced lymphocyte stimulation test, DLST) is widely used for detecting DIA, studies have reported that this test is not sufficiently sensitive for the identification of causative drugs in DIA and is affected by variations in the timing of examination [10–12]. Furthermore, LTT needs an incubation period of more than 72 hr to obtain the test sample. Therefore, it is not suitable for the rapid diagnosis of DIA apart from the determination of the causative medicine. In clinical practice, it is important to determine quickly whether the cryptogenic allergy-like symptoms are associated with DIA.

Skin tests (such as the prick test, scratch test, intradermic test, and patch test) are used for predicting DIA before drug administration [13–15]. These conventional *in vivo* tests, however, lack accuracy in risk prediction. Additionally, if positive, they are burdensome on patients because they potentially cause serious allergic symptoms such as anaphylactic shock. On the other hand, the human leukocyte antigen (HLA) genetic test has recently gained attention as a promising method to avoid DIA; however, it is necessary to determine the number of patients who developed allergy from a specific drug to identify the candidate gene [16–18].

In the present study, we report a novel method termed high-sensitive allergy test (HiSAT) that detects the humoral factors including cytokines/chemokines released from the immune cells during allergic responses by analyzing the human granulocyte-rich cell kinetics. HiSAT measures cell mobility against chemotactic factors in a gradient field of the test sample.

## Materials and methods

### Patients

Twenty-seven patients suspected of having cryptogenic allergy-like symptoms had the LTT at Fukuoka University Hospital (Fukuoka. Fukuoka, Japan) from April in 2011 to December in 2017, and the volunteers were enrolled in this study. Inclusion or exclusion criteria were patients for whom attending physician was aware of the needs for HiSAT or who had difficulty with blood sampling. The volunteers were the hospitalized patients without allergic symptoms in Fukuoka University and signed the consent form to use wasted blood of regular blood sampling. This study was approved by the Institutional Review Board of Fukuoka University Hospital (approval no. IRB11-4-16). Additionally, written informed consent was obtained from each patient before enrolment.

### Reagents

The medicines that used when cryptogenic allergy-like symptoms in individual patients occurred are summarized in S1 Table. All reagents used in this study were cell culture grade or reagent special grade.

### Cells

Peripheral blood mononuclear cells (PBMCs) were extracted from whole-blood samples of the volunteers using density-gradient centrifugation with Lymphoprep solution (AXS, Oslo, Norway), according to the manufacturer’s protocol. After washing with Hank’s balanced salt solution (HBSS; Sigma–Aldrich, St. Louis, MO, USA), the cells were resuspended at a concentration of 1.25 × 10^6^ cells/mL in RPMI 1640 medium supplemented with horse serum (5%), penicillin (100 U/mL), and streptomycin (100 μg/mL) (Sigma–Aldrich). Granulocyte-rich cell layers, containing the chemotactic cells from the blood samples of volunteers without allergy, were separated using Lymphoprep and were suspended in culture medium at a concentration of 1 × 10^7^ cells/mL.

### Sample preparation

To prepare the antigenic drug solution for HiSAT, the serum was separated from the whole blood by centrifugation for 10 min at 350 × g, after the addition of a serum-separating agent and incubation at 25˚C. The drug solutions contained the drugs at a final concentration of one-half of the C_max_ except anticancer drug or herbal medicine (S1 Table). Antigen solutions were prepared by mixing the drug solution (50 μL) with serum or culture medium (50 μL). Instead of the antigen solution, 50 μL of HBSS and PHA (Wako Pure Chemical Industries, Inc., Osaka, Japan), at a final concentration of 1 μg/mL, was used as the negative and positive controls, respectively. In 24-well plates, the PBMCs were stimulated by the addition of an antigen solution (100 μL) and subsequently cultured for 48–72 hr at 37°C in a CO_2_ incubator. The supernatants were collected and stored at -20°C until further use.

### LTT

LTT was performed by an external clinical laboratory company (SRL Inc., Hino, Tokyo, Japan). Briefly, the tritium uptake associated with T lymphocyte proliferation by PHA or antigen stimulation were measured.

### Determination of cytokine concentration in samples using flow cytometry

Cytokine/chemokine concentrations in samples were determined using FACSVerse with CBA human Th1/Th2/Th17 cytokine kit (#560484, BD) and chemokine kit (#552990, BD), according to the manufacturer’s protocol. Briefly, the mixture of human cytokine capture beads in an assay tube was incubated for 30 min at 25˚C, protected from light, after adding the Serum Enhancement Buffer. The patients’ serum, the culture supernatant, and the serial dilution of standards were then added into the capture bead mixture with PE Detection Solution. After incubating the tubes for 3 hr at 25˚C, protected from light, the beads in the assay tube were washed with 1 mL of Wash Buffer and centrifuged at 200 x *g* for 5 min. After aspirating and discarding the supernatant, 300 μL of Wash Buffer was added into each assay tube to resuspend the bead pellet, and subsequently, the acquired samples and the standards were analyzed using flow cytometry. The data of fluorescent intensity were acquired using BD CellQuest software, and subsequently, the data acquired were analyzed using FCAP array software (BD) to determine the cytokine concentrations (pg/ml) in the serum samples and the culture supernatants.

### Real-time monitoring of cell migration in HiSAT

Cell migration after exposure to sera or culture supernatants from antigen-stimulated lymphocyte cultures was recorded over time using EZ-TAXIScan® (GE healthcare, Hino, Tokyo, Japan), an optically accessible chemotaxis apparatus as described previously, with minor modifications [19–22]. Briefly, the chemotactic cells were loaded into the lower (front) wells at 5–10 μL/well and were shifted near the observation area by aspiration. Subsequently, the serum or culture medium (1.5 μL/well) was added to the upper (back) wells with a microsyringe, and a gradient was produced (gradient length, 250 μm). The observation area was 4 μm in depth for human granulocyte-rich cells. The images of chemotactic cells in the observation area were recorded after 2-min intervals over the 240-min experimental period using a computer-connected microscope. For determining cell migration velocity and the distance travelled, 10–16 migrating cells were randomly selected and the data were analyzed using ImageJ software (Wayne Rashband, Bethesda, MA, USA).

### Inhibition assay of cell migration in HiSAT using the antibody specific for cytokine receptors

The antibodies specific for the human cytokine/chemokine receptors were purchased from Sigma (anti-CXCR1, c-6223; anti-CXCR3, c-6473; anti-IL-2Rγ, i-5902) or SantaCruz (anti-IL-1R1, sc688; anti-IL-8RA, sc-7303; anti-IL-8RB, sc-32780; anti-LTB4R, sc-34348). The cells in the granulocyte-rich layer from a volunteer (chemotactic cells) were treated with 1 μg/mL of the antibody specific for the cytokine receptor for 1 hr before HiSAT. The cells were then assembled to the lower well as described above. The culture supernatant with positive control was applied to the upper well. Cell migration was sequentially recorded, and the kinetic parameters of the cell migration were analyzed using ImageJ software as mentioned above.

### Statistical analysis

Numerical data are presented as the mean ± standard error (SE). Significant differences between different treatment groups were analyzed using one-way analysis of variance, followed by Bonferroni’s multiple comparison tests or Student’s *t*-test using GraphPad Prism software (GraphPad Software Inc., La Jolla, CA, USA). *P*-value < 0.05 was considered to indicate statistical significance.

## Results

### Rapid diagnosis was enabled using HiSAT with serum

**Fig 1:**
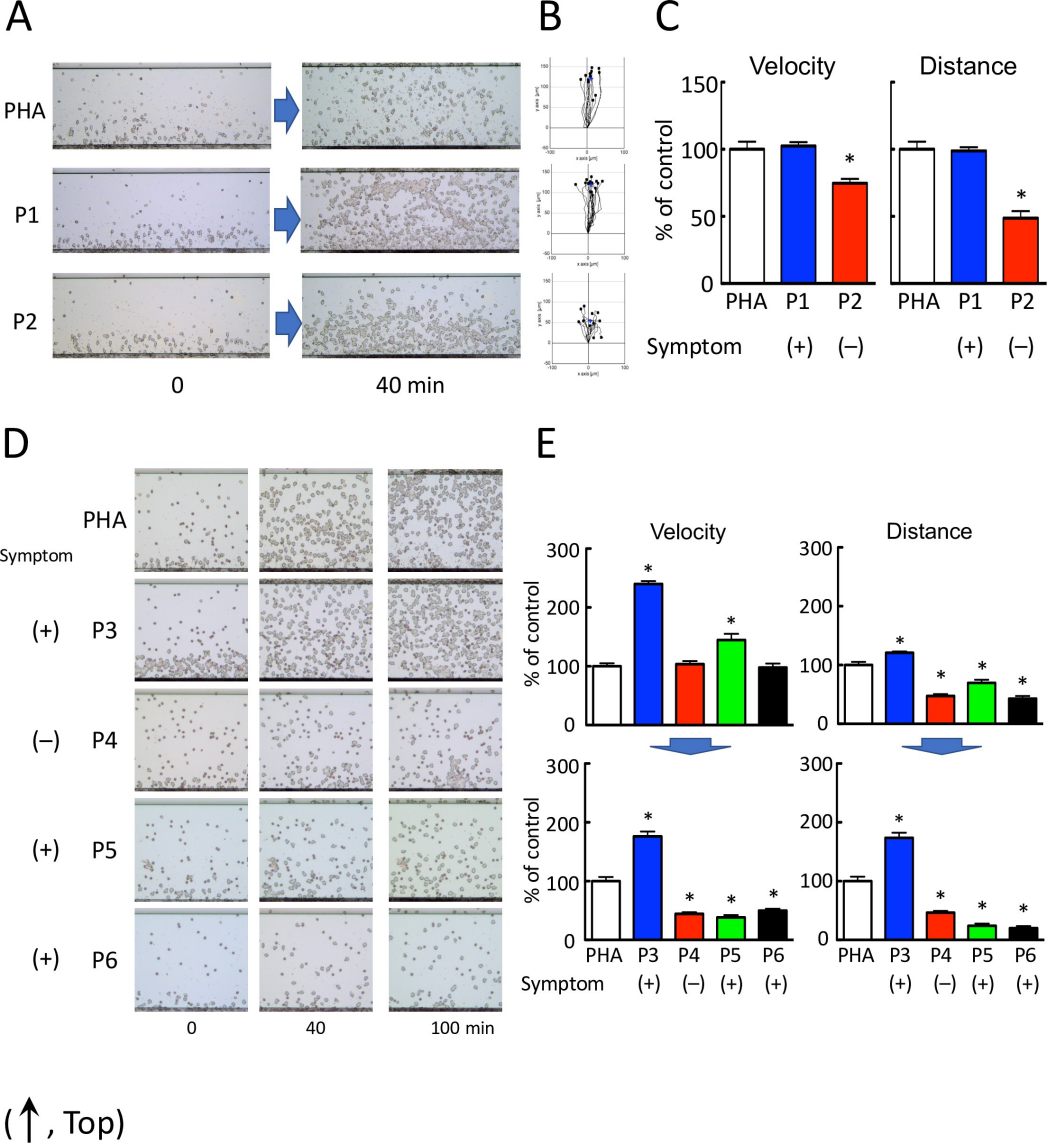
HiSAT enables the diagnosis of allergy using a patient’s serum. (A) Images of chemotactic cells in HiSAT in a patient with (P1) or without (P2) allergy-like symptoms (phytohemagglutinin [PHA], positive control consisting of the culture supernatant of a volunteer’s lymphocytes incubated with PHA). Serum from patient P1 and the culture supernatant of PHA showed significant migration of the lymphocytes compared to the serum from a patient without allergic symptoms (P2). (B) Cell migration path tracing for 40 min using ImageJ software. (C) Results of the kinetic analysis of chemotactic cells in the serum of patients P1 and P2 in HiSAT. In the serum of the patient without allergic symptoms (P2), both velocity and distance of migration of the chemotactic cells were significantly lower than those of P1 and PHA. *p < 0.001, compared with control (PHA). (D) Images of the chemotactic cells in the serum of patients P3–P6 and PHA in HiSAT. During blood sampling, patient P5 did not display any allergy-like symptoms. Human granulocytes seldom migrated against the serum of patients P4, P5, and P6 and did not show considerable differences in their mobility after 40 and 100 min. (E) HiSAT results of the kinetic analysis using the patient’s serum and PHA. Granulocytes did not migrate in 100 min post-exposure to serum samples from patients P4, P5, and P6. *p < 0.001, compared with control (PHA).

The advantages of HiSAT are its rapidity and highly sensitive quantification while assessing the serum of patients with cryptogenic allergy-like symptoms. To measure the specificity of HiSAT, we assessed the serum from patients with or without typical allergic symptoms at the time of blood sample collection. Representative images of cell migration in the serum from patients with or without allergic symptoms subjected to HiSAT are shown in Fig 1A–1C.

Granulocyte-rich cells (chemotactic cells) from volunteers migrated (velocity and distance) significantly faster when treated with the serum from a patient with nephropathy (P1) than those from a patient who had recovered from rash (P2). The chemotactic factors in the serum from a patient with allergy symptoms resulted in more rapid migration of granulocytes than those from a patient without allergy symptoms. Cell migration images of chemotactic cells treated with the serum of four patients with (P3, P4, and P6) or without (P5) cryptogenic allergic symptoms at the time of blood sampling subjected to HiSAT are shown (0–100 min, Fig 1D and S1–S5 Videos). In patients P4 and P6 suffering from pancytopenia, the allergy-like symptoms were not due to DIA. This diagnosis was supported by the clinical course of the disease. Interestingly, the granulocyte-rich cells treated with the serum of patient P3 with severe eosinophilia migrated continuously for up to 4 hr. In contrast, both the migration velocity and distance travelled by the chemotactic cell treated with the serum samples from patients P4, P5, or P6 after 100 min were markedly reduced compared to those at an earlier time point (Fig 1D and 1E).

Table 1 shows the results of HiSAT as a diagnostic method using the serum of patients with or without allergy-like symptoms at the time of blood sample collection.

**Table 1:** Results of HiSAT and LTT (DLST) in patients with allergy-like symptoms.

HiSAT				LTT (DLST)
Symptom	Rapid diagnosis*		Identification of the causative drug	
	With symptoms	Without symptoms		
Rash	–	0/8	5/8	1/6
Organopathy	6/8	0/1	3/7	0/2
Eosinophilia	1/2	–	3/3	1/1
Interstitial pneumonia	1/1	–	1/1	0/1
Other	0/3	1/2	0/1	–
Total	8/14 (57%)	1/11 (9%)	12/20 (60%)	2/10 (20%)
After exclusion of non-allergy patients	8/9 (89%)	1/11 (9%)	–	–

*Patient displayed allergy-like symptoms during blood sampling [indicated by “with symptoms”]

Eight out of 14 serum samples from patients with allergy-like symptoms were HiSAT-positive. In contrast, 10 out of 11 serum samples without allergy-like symptoms was HiSAT-negative. According to their clinical course, cytopenia (three samples) and hepatic disorders (two samples) were not characterized as allergies. After excluding the false-positive cases, the true-positive rate of diagnosis by HiSAT was 89% (8/9) for patients with allergic symptoms, and 9% (1/11) for those without allergic symptoms.

### Cytokine concentration in serum did not necessarily reflect allergic symptoms in patients

The standard curve was obtained for each cytokine using the series of standard, and the determination coefficient (r^2^) of the cytokines was calculated over 0.999, except IL-17A and MCP-1 (0.998), as shown in S1 Fig.

**Fig 2:**
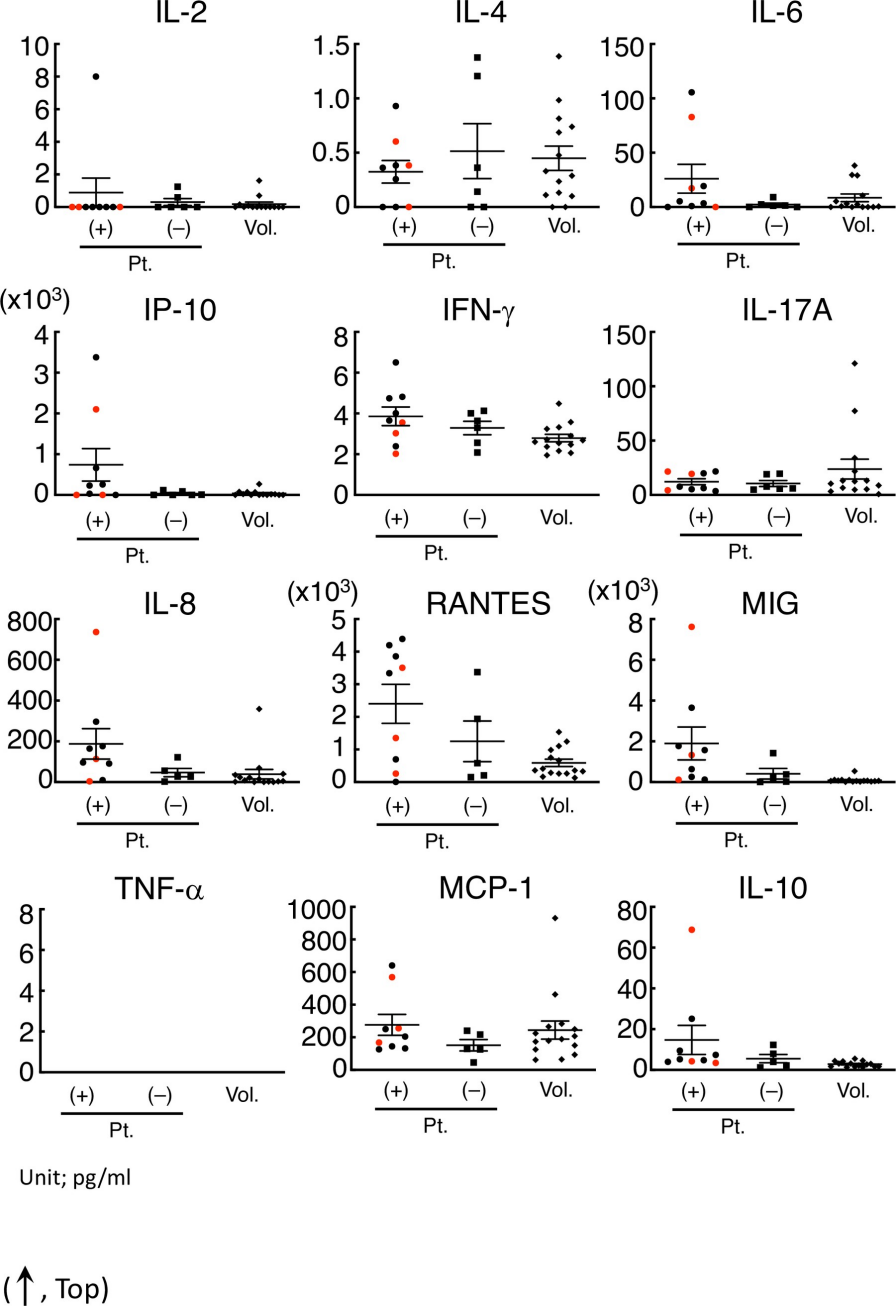
IFN-γ, IL-8, RANTES, and MIG in the serum of patients with allergic symptoms were significantly higher than those in volunteers. The concentration of IFN-α (omitted) and IL-2 in the serum of most subjects was out of range in our condition. The IP-10, IFN-γ, IL-17A, IL-8, RNATES, and MIG concentrations in the serum of the patients with allergy-like symptoms were significantly different from those of volunteers. Even in those cytokines, the concentration in the serum from patients with symptoms was not necessarily higher than that from volunteers or patients without symptoms. The red points in each cytokine were regarded as those from non-allergy patients diagnosed using clinical records.

In this study, the concentration of IL-4, IL-6, IFN-γ, TNF-α, IL-17A, IL-8, RANTES, MCP-1, and IL-10 in the serum of patients or volunteers was successfully determined, whereas the concentration of IL-2 and TNF-α in the serum, especially of volunteers, was too low to determine (Fig 2).

The three red points in the serum of patients with cryptogenic allergy-like symptoms indicated that it was non-allergy according to the clinical course. After omitting the non-allergy cases, four cytokines (IFN-γ, IL-8, RANTES, and MIG) in the serum of patients with allergic symptoms were significantly higher than those in volunteers (Table 2).

**Table 2:** Comparison of cytokine concentration (pg/ml) in the serum between allergy patients and volunteers.

Cytokine	Allergy patients	Volunteers
IL-2	1.14 **±** 3.03	0.183 **±** 0.454
IL-4	0.322 **±** 0.343	0.449 **±** 0.417
IL-6	22.4 **±** 41.3	8.54 **±** 13.3
IL-10	15.22 **±** 26.08	0.787 **±** 1.43
IFN-γ*	4.35 **±** 1.38	2.79 **±** 0.679
IL-17A	10.6 **±** 7.47	23.8 **±** 34.1
IL-8*	138 **±** 97.7	39.0 **±** 90.7
RANTES*	27478 **±** 16516	5896.2 **±** 4376.5
MIG*	1334.4 **±** 1320.1	91.7 **±** 127
MCP-1	249 **±** 197	244 **±** 215
IP-10	466 **±** 399	140 **±** 67.0

*p < 0.01, indicates significant difference between patients and volunteers

On the other hand, the concentration of these cytokines in some patients with symptoms was lower than that in patients without symptoms or in volunteers (Fig 2).

### Causative drug of allergy was determined by HiSAT using culture supernatant with higher accuracy than LTT

**Fig 3:**
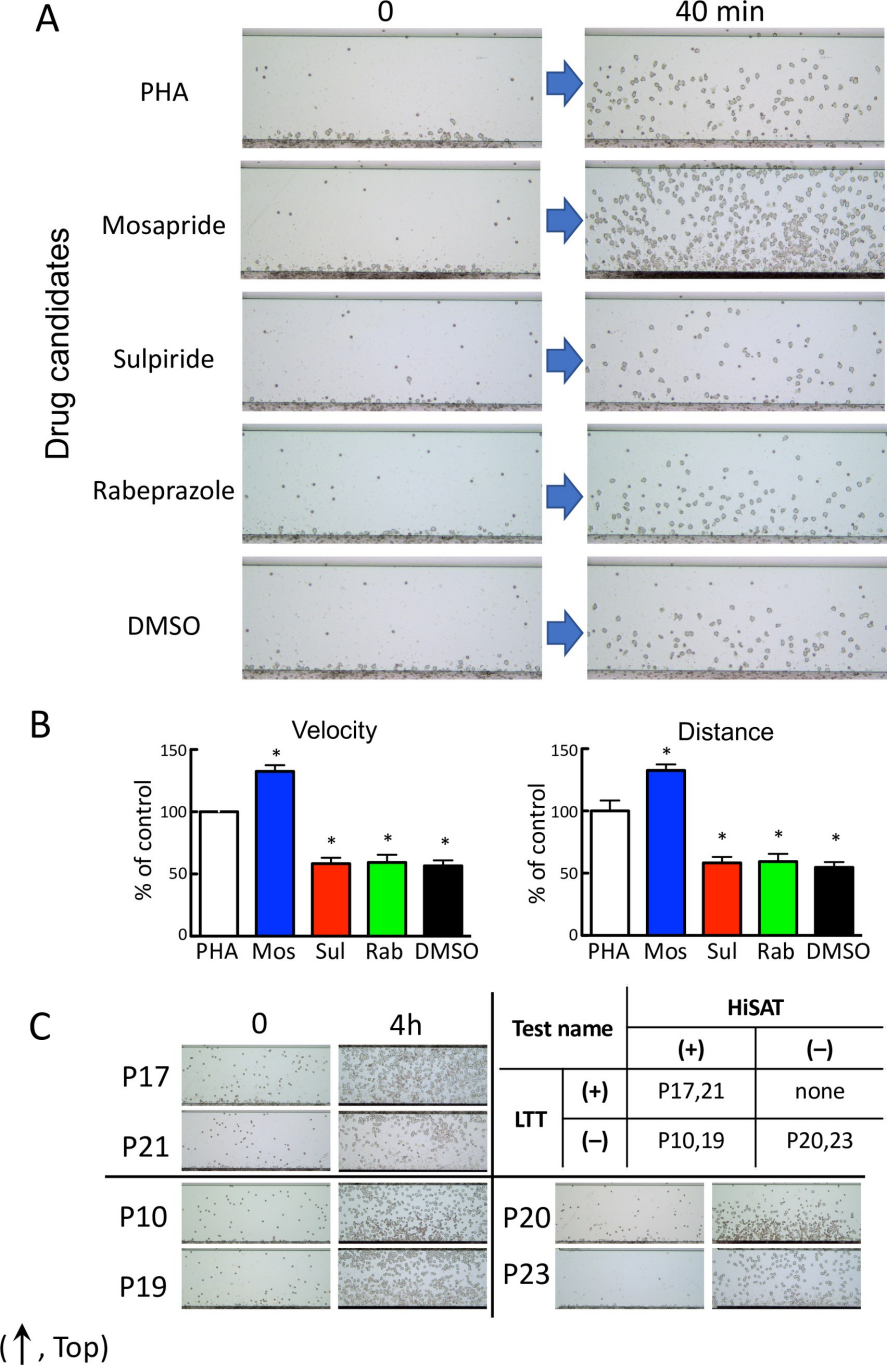
Determination of the causative drug for DIA (in patient P3) using HiSAT. (A) Images of chemotactic cells in the culture supernatants of P3 lymphocytes incubated with three candidate drugs in HiSAT. The lymphocytes in the culture supernatant of P3 incubated with mosapride for 72 hr displayed increased mobility compared to that of PHA. DMSO was used as a solvent for dissolving the hydrophobic drugs. (B) Results of the kinetic analysis for candidate drugs causing DIA using HiSAT. At earlier time points, mosapride treatment significantly increased the migration (velocity and distance) of granulocytes. *p < 0.001, compared with control. (C) Difficulty in the determination of the causative drug using start and end point images in HiSAT. Upper right, the results of the LTT and HiSAT to determine the causative drug in patients with allergy-like symptoms. LTT-positive and HiSAT-negative cases were not observed in this study. (The images were the end points in HiSAT, using the culture supernatant of lymphocytes from patients (P10, P17, P19, P20, P21 or P23) and the candidate drug). Upper left, HiSAT and LTT double-positive cases. Lower left, HiSAT positive but not LTT cases. Lower right, HiSAT and LTT double-negative cases.

HiSAT was performed using the culture supernatant of the lymphocytes from patient P3, which were incubated with an antigen (the candidate drug) to detect the drug responsible for DIA. Granulocyte-rich cells from volunteers treated with the culture supernatant incubated with the lymphocytes of P3 and mosapride (the antigen solution), one of the three candidate drugs, showed significantly higher migration than that of phytohemagglutinin (PHA; Fig 3A–3C).

In Fig 3C, the migrating cell images at the end point of observation (4 hr) were assessed to diagnose DIA using the samples with positive and negative results in both HiSAT and LTT. Because granulocyte-rich cells from the volunteers had different reactivity to the test sample, it was difficult to diagnose using only the images at the end point.

### Cell mobility toward culture supernatant of lymphocytes in pollinosis patient and pollen extracts increased in a dose-dependent manner in HiSAT

**Fig 4:**
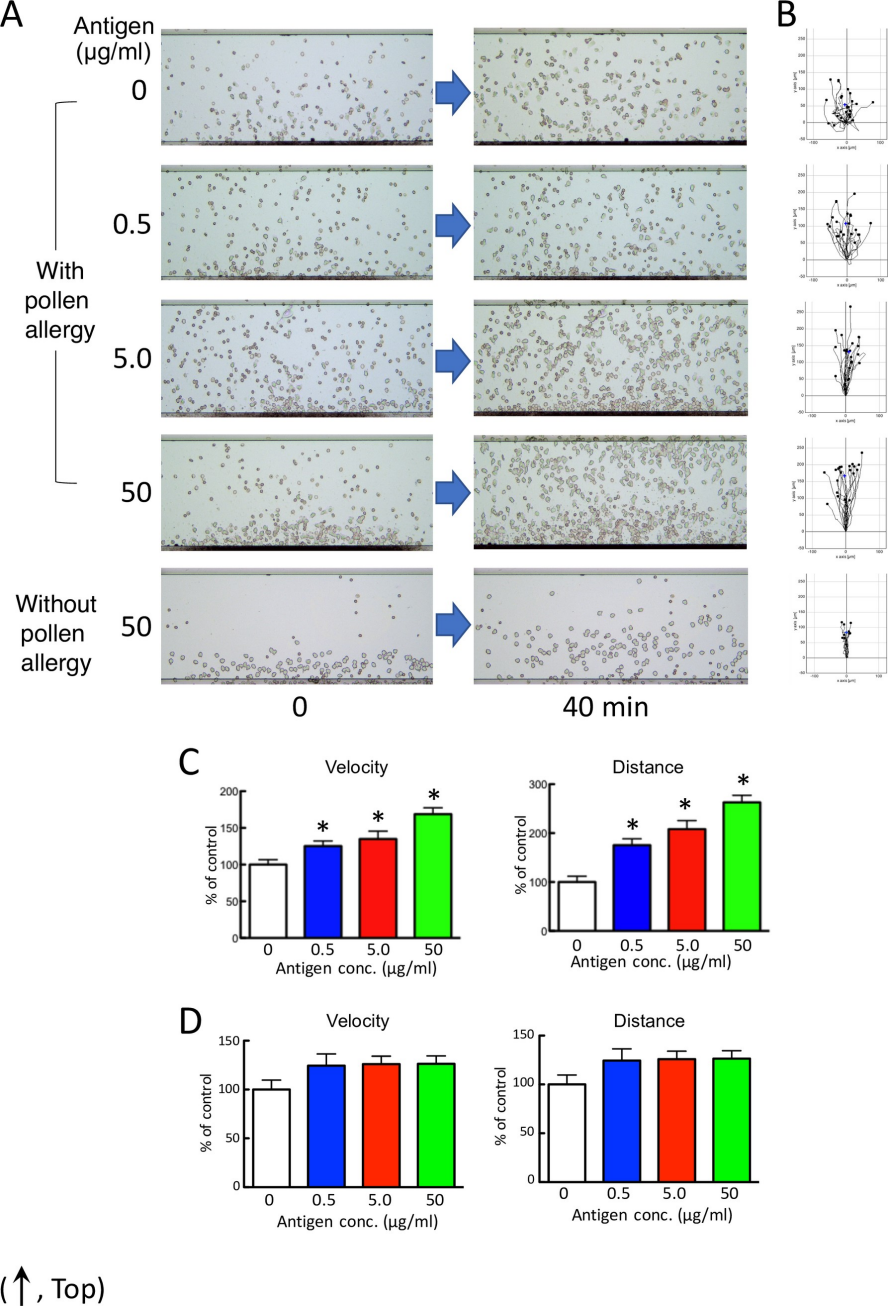
Human granulocytes migrate in an antigen dose-dependent manner against the culture supernatant. (A) Chemotactic cell images by HiSAT in subjects with or without cedar pollen allergy. After 40 min, more migrating cells are observed on the stage in the supernatant incubating lymphocytes from a subject with pollinosis with a higher dose of antigen than from a subject without pollinosis. (B) Cell migration path tracing for 40 min using ImageJ software. (C and D) The results of the kinetic analysis using HiSAT in the sample incubating lymphocytes from a subject with (C) or without (D) cedar pollen allergy and cedar pollen extract. Granulocytes (chemotactic cells) from a volunteer migrated only in a dose-dependent manner (velocity and distance) in samples with pollinosis. Data are presented as the mean ± SE (n = 10–18). *p < 0.001, compared with control (without antigen).

Granulocyte-rich cells from volunteers showed increased cell mobility (velocity, distance) in an antigen dose-dependent manner against the culture supernatant when incubated for 48 hr with cedar pollen extracts and the subject’s lymphocytes with cedar pollen allergy, without allergic symptoms at the time of sample collection (Fig 4A–4C and S6–S10 Videos).

### Cytokine concentration profile that evoke cells migration depends on the stimulating antigen

**Fig 5:**
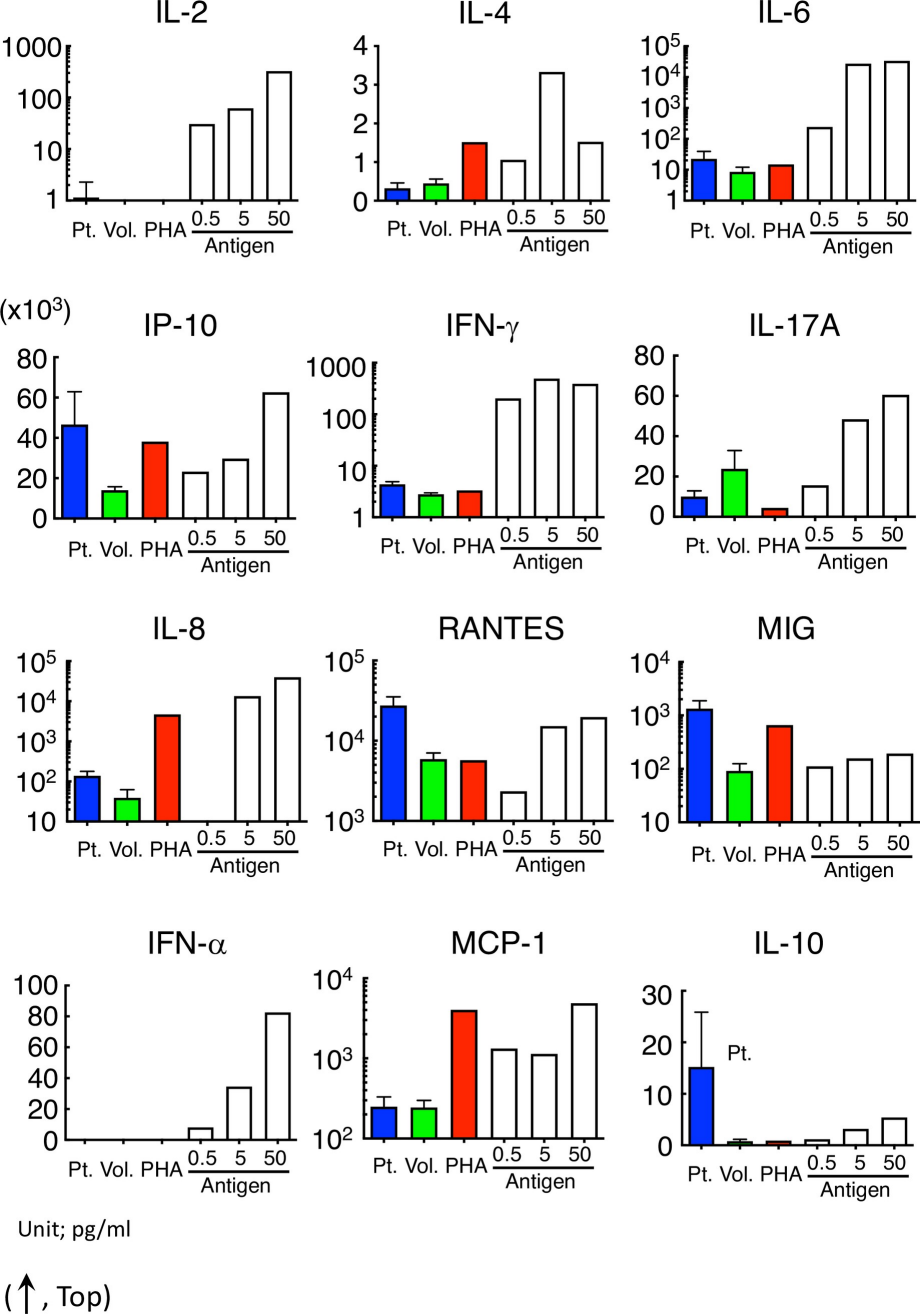
Cytokine released from lymphocytes was different among allergy patients, PHA-stimuli and pollinosis. Cytokine concentration in the serum of patients or in the culture supernatant incubated with PHA or pollen extract with the lymphocytes of volunteers or pollinosis patients revealed no specific tendency. Concentrations of IL-2 and IFN-α were out of range except with pollinosis.

In this study, the lymphocytes of subjects were artificially stimulated by PHA or cedar pollen extract. Cytokine production in the serum sample of the subjects or the culture supernatant was different by the kind of ligand that stimulated the lymphocytes, as shown in Fig 5.

The IP-10, IL-8, and MIG tended to be high both in the serum of allergy patients and in the supernatant after the artificial stimulation of lymphocytes. Although both IFN-γ and RANTES were significantly high in the serum of patients with allergic symptoms, their concentrations after PHA stimulation were similar to those in the volunteers. The cytokines were released in an antigen dose-dependent manner by stimulating with cedar pollen extracts similar to the cell migration in HiSAT.

### Antibody specific for the cytokine receptor inhibited cell migration in HiSAT

**Fig 6:**
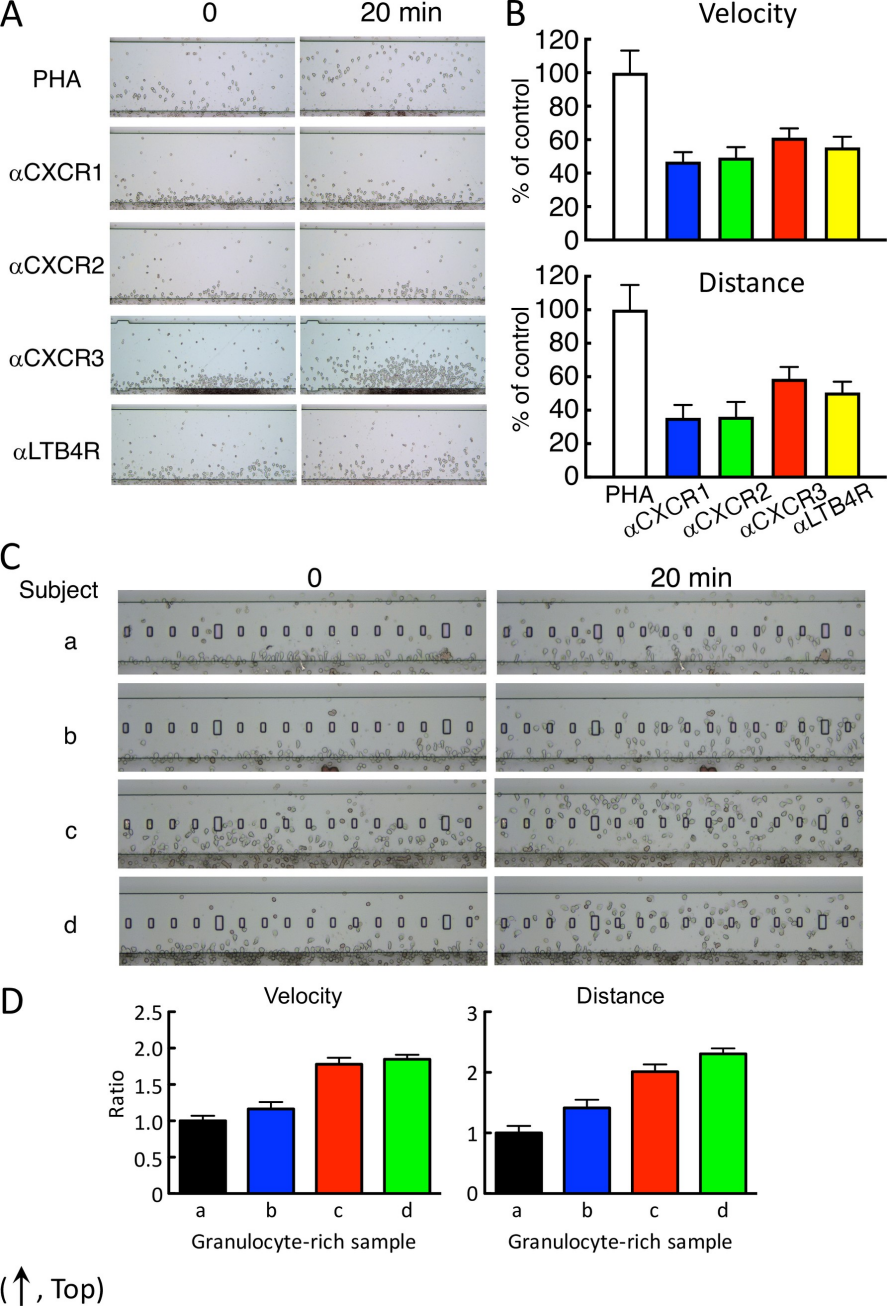
Cell mobility were prevented by antibody specific for IL-8R and LTB4R and depended on the individuals. (A) Typical cell images in the prevention of cell mobility by the specific antibody in HiSAT. Anti-IL-8R or anti-LTB4R antibody inhibited cell migration to the PHA culture supernatant after 20 min. (B) Both velocity and distance were significantly reduced by the application of the specific antibody in HiSAT. (C) Different chemotactic cell mobility against the same positive culture supernatant in four individual volunteers. Images of the granulocyte-rich layer from four volunteers (a–d) were indicated at 0 and 20 min after observation, respectively. Cells from c and d were across the observation stage after 20 min, whereas cells from a and b reached half of the stage. (D) Cell kinetics were different in the four individuals. Cell mobilities against the same culture sample were different approximately two or more folds (velocity or distance, respectively) from the individuals.

We assessed whether cell migration was affected by covering the cytokine receptor with the specific antibody. As expected, anti-IL-1R and anti-IL-2R were not affected by their specific antibodies, whereas anti-IL-8R or anti-LTB4R2 antibody significantly prevented cell migration against PHA-stimulated supernatant in HiSAT (Fig 6A and 6B).

### Granulocyte-rich cell reactivity from different individuals against chemotactic factors

Based on their mobility and sensitivity, the human granulocyte-rich cells were used as migrating/chemotactic cells for HiSAT in this study. However, the reactivity and/or sensitivity of chemotactic cells in different individuals were at least twofold different (Fig 6C and 6D). Although the Jurkat cells and other established cell lines also migrated toward chemotactic factors in samples, their sensitivity was <20% compared to human granulocyte-rich cells under the conditions used in the present study (S2 Fig).

## Discussion

In this study, it was demonstrated that HiSAT, used to analyze the cell kinetics in a gradient field of humoral chemotactic factors in the patients’ serum or in the culture supernatant incubated with the patient’s lymphocytes and an antigen solution, is a promising method for the rapid diagnosis and determination of the allergy-causing antigen (S3 Fig). It can also be used for the risk prediction before drug administration. The advantages of using HiSAT over the conventional methods are as follows: (i) it is a simple method that can be used in three different ways depending on the clinical situation. (ii) It minimizes invasiveness in patients because of the small sample size required for analysis. (iii) It is highly sensitive and useful for demonstrating the antigen dose-dependency. (iv) It is useful for assessing the combination of multiple chemotactic factors including cytokines/chemokines required for the induction of cell migration in allergy (S4 Fig).

Allergic reactions including DIA are frequently classified into four immunological categories [23, 24]. The conventional allergy tests measure the type-specific antibodies (such as IgE–radioallergosorbent test and multiple allergosorbent test system), effector/chemical mediators (such as histamine release test), or T cell proliferation (such as LTT/DLST). However, the application of HiSAT is not limited to a certain type of allergic reaction. During an allergic response, some reactions are well characterized, but other molecular mechanisms or signaling cascades remain unknown or poorly understood. In this context, HiSAT, which is used to analyze the humoral factors including cytokines/chemokines and their combinations during allergy in individual patients, is a promising method for allergy testing. The concentrations of cytokines or chemokines in serum have been determined in patients with or without allergic symptoms. However, there are still considerable debates, and there is no consensus thus far. As shown in Fig 2 and Table 2, four cytokines (IFN-γ, IL-8, RANTES and MIG) were significantly higher in allergic patients, and they would be potential markers for allergic patients. However, the cytokine concentration in patients with allergy-like cryptogenic symptoms is not appropriate to diagnose DIA, because it is not necessary for the concentration to be higher than those in volunteers or in patients without symptoms. Furthermore, the inhibitory assay using specific antibody might support the importance of the combination of cytokine for the DIA symptom. Some cytokines such as IP-10 and IL-8, after the artificial stimulation with PHA or cedar pollen extract, had a similar pattern but other cytokines such as IFN-γ, IL-17A and MIG did not under our experimental condition (Fig 5). This result also indicates that it is difficult to diagnose DIA using the cytokine concentration [25, 26]. Interestingly, the combination of these antibodies strongly inhibited cell migration. As IL8 is high in the supernatant that is artificially stimulated by PHA or cedar pollen extracts, it seems to play an important role. However, considering that it is not necessarily high in patients’ sera, and its inhibition by specific antibodies was approximately 50%, coordination with other cytokines or their combination might be more important (Fig 6A and 6B).

In clinical practice, rapid diagnosis of the cryptogenic allergy-like symptoms is required, as DIA interrupts the treatment of the primary disease. Considering the high positive-detection rate of HiSAT (excluding the non-allergic cases), it is an easy and rapid (time required, 1 hr) tool for diagnosing DIA in clinical practice using a small amount (~2 μL) of the patient’s serum (S4 Fig). However, further studies are required to confirm the universal applicability of this method. It is notable that two hepatic disorders tested negative for HiSAT, implying that the non-allergic inflammation might become negative and can be distinguished from the allergic one.

Conventional allergy tests (such as LTT) widely used for determining the causative drugs for DIA lack accuracy [10, 27–30]. In this study, LTT was used to determine the causative drug for DIA in only 20% of the samples, whereas HiSAT was used to determine the causative drug with a threefold higher efficiency. If the rapid diagnosis using HiSAT is performed in advance, followed by LTT, then the positive-detection rate of the causative drug for DIA might increase.

HiSAT might theoretically predict the potential allergic reactions in response to an antigen, even if no allergy-like symptoms were observed. Importantly, granulocyte-rich cells, such as the chemotactic cells used for performing HiSAT, were obtained from a volunteer without allergy but not from a patient with allergic symptoms. This can also minimize invasiveness in patients during blood sampling for HiSAT. Furthermore, it measures not only qualitative reactivity similar to the conventional tests but also the quantitative reactivity (such as antigen dose-dependency), by incubating the patient’s lymphocytes with the antigen. As for the antigen dose-dependency of HiSAT, the extent of chemotactic cell reaction might indicate the severity of allergy and determine the threshold dose for the induction of allergy in different individuals. Importantly, it was possible to detect significant differences in cell migration, as compared with the control, by analyzing the imaging data that were obtained in less than 1 hr, by incubating the lymphocytes of subjects’ with higher concentrations of the cedar pollen extract for 24 hr in HiSAT as in patient B (Fig 4). Therefore, HiSAT might be expected to rapidly determine the causative drug in patients with a serious presentation or severe symptoms.

Owing to the ease of handling and differential reactivity of the human granulocyte-rich cells from different individuals (Fig 6C), it might be necessary to investigate the most appropriate chemotactic cells (such as Jurkat cells) for obtaining reproducible results in HiSAT. It is not surprising that individual cells have different reactivity to the culture supernatants stimulated by antigens, given the previous reports of altered chemotactic ability to chemotactic factors in the cells of allergy patients [31]. In allergy patients, it is thought that in addition to changes in the cytokine profile in the blood, the responsiveness to cytokines also changes, which may be one of the reasons why it is difficult to diagnose allergy or identify the causative drug. Although the cell lines established using human hematological malignancies have demonstrated less reactivity, some cell lines displayed enhanced migratory abilities after differentiation induced by dimethyl sulfoxide (DMSO) or all-trans retinoic acid. Thus, it may be possible to establish new cell lines by transfecting and expressing the appropriate genes in them.

## Conclusions

HiSAT is a promising method for risk prediction, rapid diagnosis, or determination of the antigen responsible for causing allergy. However, further investigation is required to confirm the universal applicability of HiSAT for all types of allergy reactions. A clinical study to establish HiSAT as a method for predicting DIA in clinical samples has been initiated.

## Supporting information

S1 Fig Dot blots, standard curve and r^2^ of cytokines in CBA kit. Typical blot pattern at 3 of 10 concentrations of cytokine standard in flow cytometry with the Cytometric Bead Array (CBA) assay kit (A), typecal standard curve (B) and the r^2^ value that was fitted and was calculated using CBA software (C). The standard curves are well fitted, and the r^2^ values are greater than 0.998 under our experimental condition.
(PDF)

S2 Fig Reactivity of Jurkat cells is lower than that of granulocytes-rich cells in HiSAT. The migration of Jurkat cells toward the culture supernatant by incubating the volunteer’s lymphocytes and phytohemagglutinin (PHA) for 48 hr in high-sensitive allergy test (HiSAT). The velocity or distance was calculated as <20% of chemotactic cells (granulocyte-rich cells) from the volunteer. A and B, typical images of Jurkat cell migration at 0 and 50 min after sample application in HiSAT. C and D, Cell migration path tracing of patient #3 and volunteer for 40 min using ImageJ software. E, The results of the kinetic analysis of Jurkat cells in the culture supernatant in HiSAT. Jurkat cells were purchased from American Type Culture Collection (ATCC). The cells were maintained in RPMI 1640 medium supplemented with fetal bovine serum (5%) (Hyclone, Cytiva), penicillin (100 U/mL), and streptomycin (100 μg/mL) (Sigma–Aldrich) at 37˚C in a CO_2_ incubator. The medium was changed twice a week. The cells were washed with PBS and subsequently used in HiSAT.
(PDF)

S3 Fig Principal of HiSAT. A novel method, high-sensitive allergy test (HiSAT), enables rapid diagnosis and determination of the antigen causing or predicting allergy, with high accuracy, by analyzing cell kinetics as an index against chemotactic factors in blood or culture samples.
(PDF)

S4 Fig Inspection flow chart of HiSAT. The advantages of HiSAT are its rapidity and highly sensitive quantification while assessing the serum of patients with cryptogenic allergy-like symptoms. For determination of causative medicine, the supernatant incubated patient’s lymphocytes with a candidate medicine is used instead of serum of patients.
(PDF)

S1 Table Details of patients and candidate medicine examined. Twenty-seven patients with cryptogenic allergy-like symptoms were examined rapid test with serum, determination of causative medicine in HiSAT or LTT.
(PDF)

S1 Video Chemotactic cells (granulocytes-rich cells) from volunteers migrated against the patient’s serum or culture supernatant of positive control (phytohemagglutinin [PHA] stimulation). The granulocyte-rich cells treated with serum from patient P3 with severe eosinophilia migrated drastically fast and continuously for up to 4 hr (S2 Video). Video image: S1 Video, PHA control; S2 Video, patient P3; S3 Video, patient P4; S4 Video, patient P5; S5 Video, patient P6.
(AVI)

S2 Video Chemotactic cells (granulocytes-rich cells) from volunteers migrated against the patient’s serum or culture supernatant of positive control (phytohemagglutinin [PHA] stimulation). The granulocyte-rich cells treated with serum from patient P3 with severe eosinophilia migrated drastically fast and continuously for up to 4 hr (S2 Video). Video image: S1 Video, PHA control; S2 Video, patient P3; S3 Video, patient P4; S4 Video, patient P5; S5 Video, patient P6.
(AVI)

S3 Video Chemotactic cells (granulocytes-rich cells) from volunteers migrated against the patient’s serum or culture supernatant of positive control (phytohemagglutinin [PHA] stimulation). The granulocyte-rich cells treated with serum from patient P3 with severe eosinophilia migrated drastically fast and continuously for up to 4 hr (S2 Video). Video image: S1 Video, PHA control; S2 Video, patient P3; S3 Video, patient P4; S4 Video, patient P5; S5 Video, patient P6.
(AVI)

S4 Video Chemotactic cells (granulocytes-rich cells) from volunteers migrated against the patient’s serum or culture supernatant of positive control (phytohemagglutinin [PHA] stimulation). The granulocyte-rich cells treated with serum from patient P3 with severe eosinophilia migrated drastically fast and continuously for up to 4 hr (S2 Video). Video image: S1 Video, PHA control; S2 Video, patient P3; S3 Video, patient P4; S4 Video, patient P5; S5 Video, patient P6.
(AVI)

S5 Video Chemotactic cells (granulocytes-rich cells) from volunteers migrated against the patient’s serum or culture supernatant of positive control (phytohemagglutinin [PHA] stimulation). The granulocyte-rich cells treated with serum from patient P3 with severe eosinophilia migrated drastically fast and continuously for up to 4 hr (S2 Video). Video image: S1 Video, PHA control; S2 Video, patient P3; S3 Video, patient P4; S4 Video, patient P5; S5 Video, patient P6.
(AVI)

S6 Video Chemotactic cells from volunteers migrated in a dose-dependent manner in HiSAT by stimulating the subject’s lymphocytes (S6–S9 Videos, the subject with cedar pollen allergy without symptom; S10 Video, the subject without cedar pollen allergy) with cedar pollen extracts for 48 hr at the indicating concentration. S6 Video, 0 μg/ml; S7 Video, 0.5 μg/ml; S8 Video, 5.0 μg/ml; S9 Video, 50 μg/ml; S10 Video, 50 μg/ml.
(AVI)

S7 Video Chemotactic cells from volunteers migrated in a dose-dependent manner in HiSAT by stimulating the subject’s lymphocytes (S6–S9 Videos, the subject with cedar pollen allergy without symptom; S10 Video, the subject without cedar pollen allergy) with cedar pollen extracts for 48 hr at the indicating concentration. S6 Video, 0 μg/ml; S7 Video, 0.5 μg/ml; S8 Video, 5.0 μg/ml; S9 Video, 50 μg/ml; S10 Video, 50 μg/ml.
(AVI)

S8 Video Chemotactic cells from volunteers migrated in a dose-dependent manner in HiSAT by stimulating the subject’s lymphocytes (S6–S9 Videos, the subject with cedar pollen allergy without symptom; S10 Video, the subject without cedar pollen allergy) with cedar pollen extracts for 48 hr at the indicating concentration. S6 Video, 0 μg/ml; S7 Video, 0.5 μg/ml; S8 Video, 5.0 μg/ml; S9 Video, 50 μg/ml; S10 Video, 50 μg/ml.
(AVI)

S9 Video Chemotactic cells from volunteers migrated in a dose-dependent manner in HiSAT by stimulating the subject’s lymphocytes (S6–S9 Videos, the subject with cedar pollen allergy without symptom; S10 Video, the subject without cedar pollen allergy) with cedar pollen extracts for 48 hr at the indicating concentration. S6 Video, 0 μg/ml; S7 Video, 0.5 μg/ml; S8 Video, 5.0 μg/ml; S9 Video, 50 μg/ml; S10 Video, 50 μg/ml.
(AVI)

S10 Video Chemotactic cells from volunteers migrated in a dose-dependent manner in HiSAT by stimulating the subject’s lymphocytes (S6–S9 Videos, the subject with cedar pollen allergy without symptom; S10 Video, the subject without cedar pollen allergy) with cedar pollen extracts for 48 hr at the indicating concentration. S6 Video, 0 μg/ml; S7 Video, 0.5 μg/ml; S8 Video, 5.0 μg/ml; S9 Video, 50 μg/ml; S10 Video, 50 μg/ml.
(AVI)
