# Supplementary figures and images for: Novel method to analyze cell kinetics for the rapid diagnosis and determination of the causative agent in allergy
Source: PLoS One. 2021 Feb 19;16(2):e0246125. doi: 10.1371/journal.pone.0246125 (PMC7895410; doi:10.1371/journal.pone.0246125)

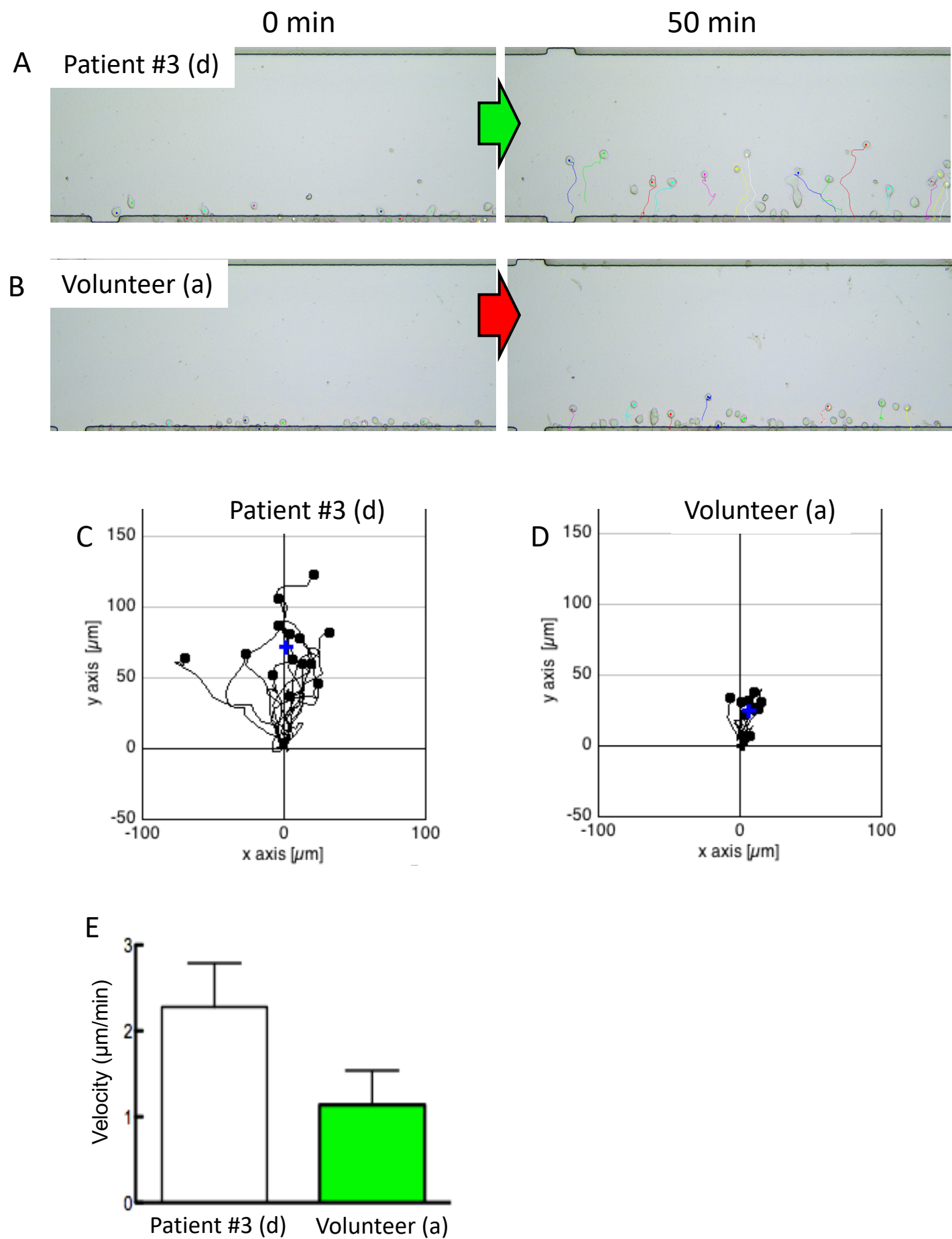

Supplement: S2 Fig — The migration of Jurkat cells toward the culture supernatant by incubating the volunteer’s lymphocytes and phytohemagglutinin (PHA) for 48 hr in high-sensitive allergy test (HiSAT). The velocity or distance was calculated as <20% of chemotactic cells (granulocyte-rich cells) from the volunteer. A and B, typical images of Jurkat cell migration at 0 and 50 min after sample application in HiSAT. C and D, Cell migration path tracing of patient #3 and volunteer for 40 min using ImageJ software. E, The results of the kinetic analysis of Jurkat cells in the culture supernatant in HiSAT. Jurkat cells were purchased from American Type Culture Collection (ATCC). The cells were maintained in RPMI 1640 medium supplemented with fetal bovine serum (5%) (Hyclone, Cytiva), penicillin (100 U/mL), and streptomycin (100 μg/mL) (Sigma–Aldrich) at 37˚C in a CO2 incubator. The medium was changed twice a week. The cells were washed with PBS and subsequently used in HiSAT. (PDF) [file pone.0246125.s002.pdf]

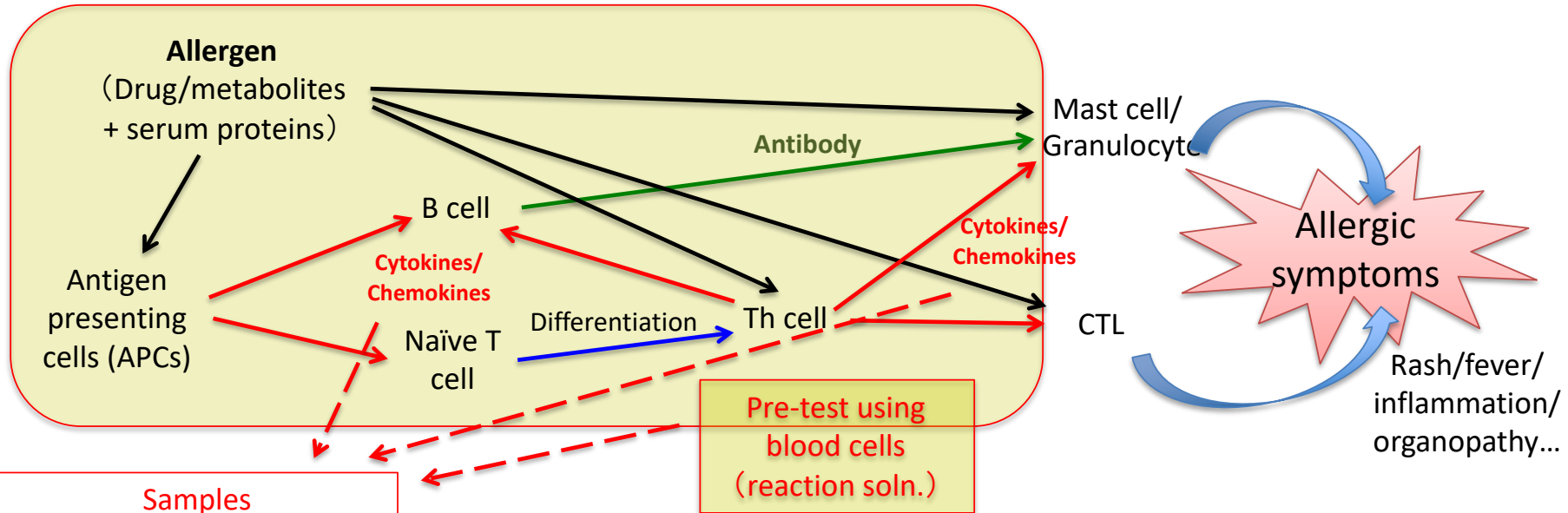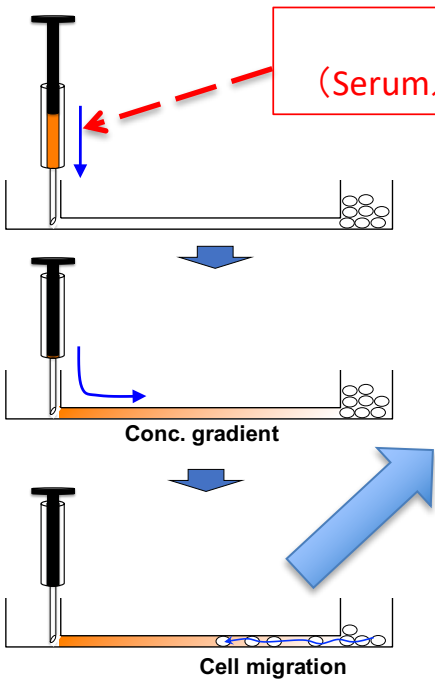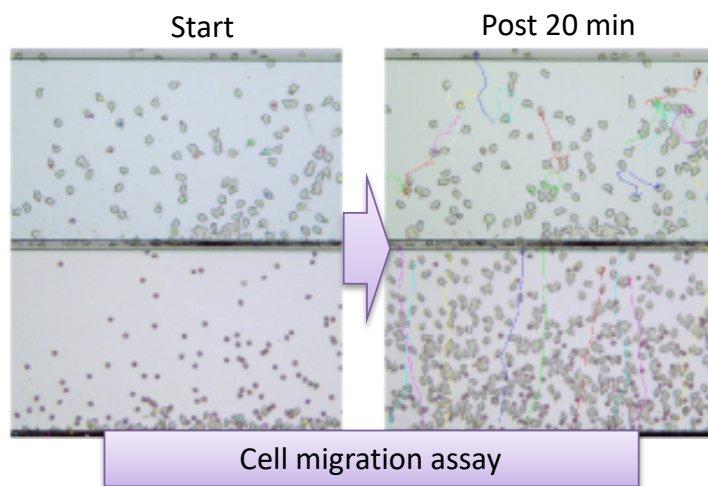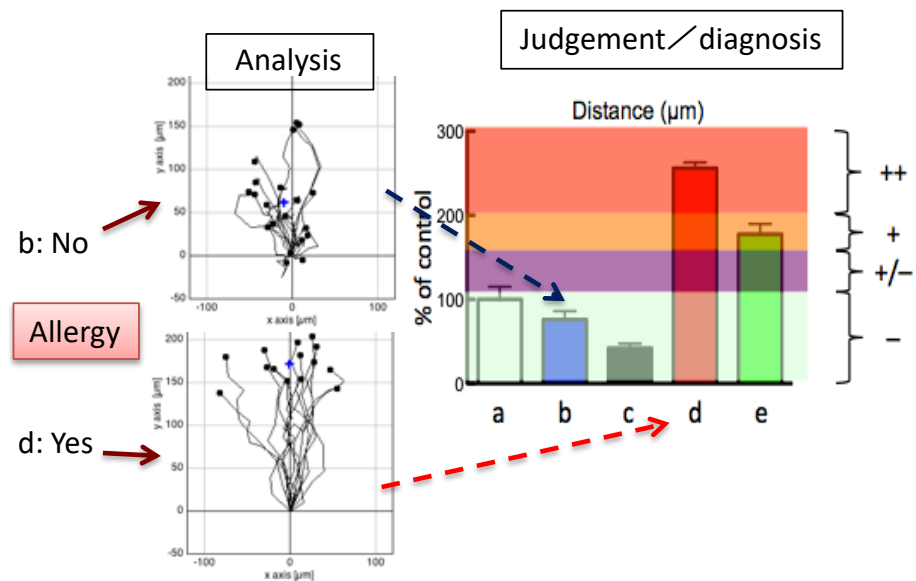

Supplement: S3 Fig — A novel method, high-sensitive allergy test (HiSAT), enables rapid diagnosis and determination of the antigen causing or predicting allergy, with high accuracy, by analyzing cell kinetics as an index against chemotactic factors in blood or culture samples. (PDF) [file pone.0246125.s003.pdf]
