# Supplementary material for: Novel method to analyze cell kinetics for the rapid diagnosis and determination of the causative agent in allergy
Source: PLoS One. 2021 Feb 19;16(2):e0246125. doi: 10.1371/journal.pone.0246125 (PMC7895410; doi:10.1371/journal.pone.0246125)
Supplement: S4 Fig — The advantages of HiSAT are its rapidity and highly sensitive quantification while assessing the serum of patients with cryptogenic allergy-like symptoms. For determination of causative medicine, the supernatant incubated patient’s lymphocytes with a candidate medicine is used instead of serum of patients. (PDF) [file pone.0246125.s004.pdf]

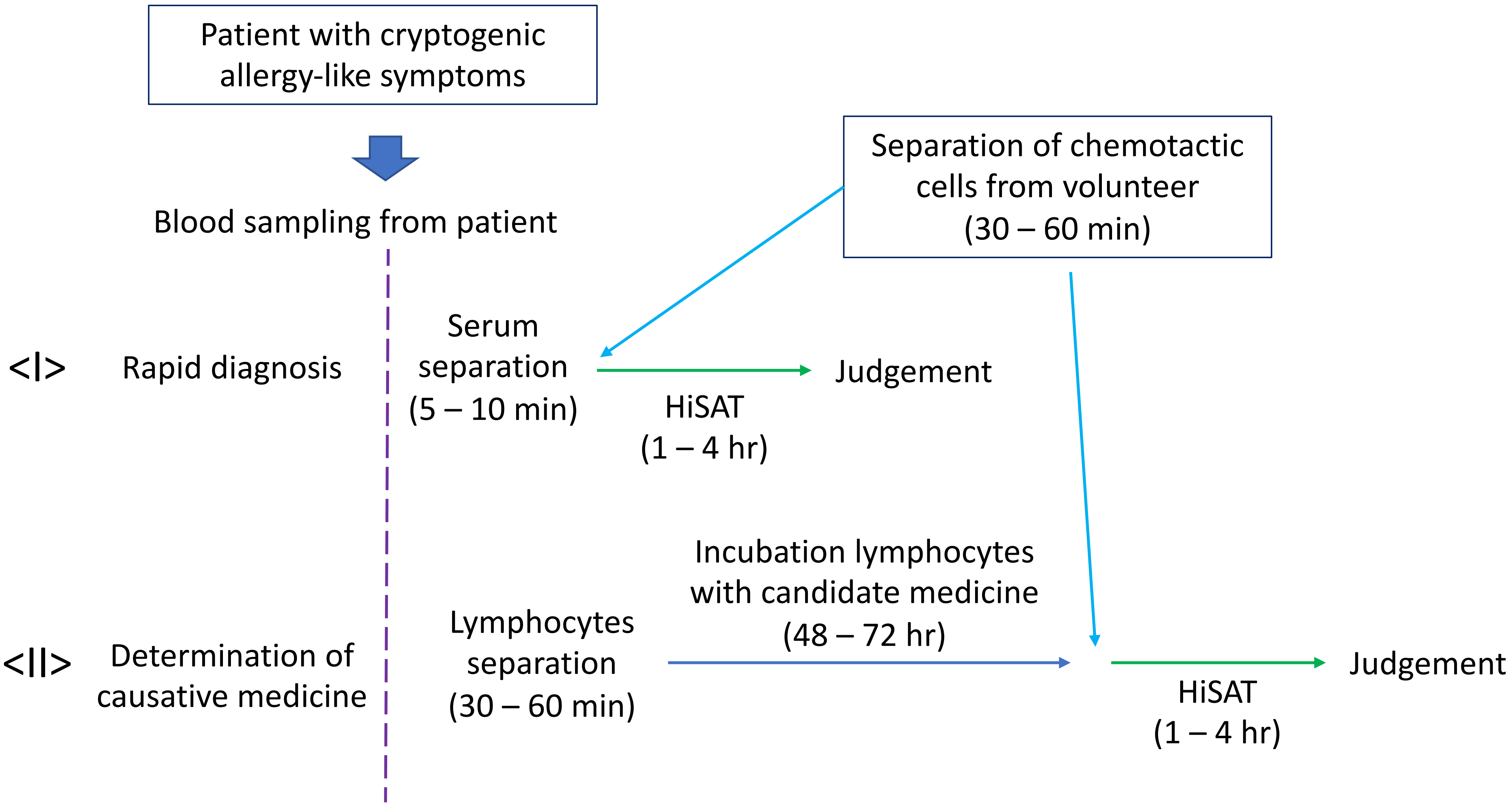
Patient with cryptogenic allergy-like symptoms
Blood sampling from patient
Separation of chemotactic cells from volunteer (30 – 60 min)
<I>
Rapid diagnosis
Serum separation (5 – 10 min)
HiSAT (1 – 4 hr)
Judgement
<II>
Determination of causative medicine
Lymphocytes separation (30 – 60 min)
Incubation lymphocytes with candidate medicine (48 – 72 hr)
HiSAT (1 – 4 hr)
Judgement
