# Supplementary material for: Novel method to analyze cell kinetics for the rapid diagnosis and determination of the causative agent in allergy
Source: PLoS One. 2021 Feb 19;16(2):e0246125. doi: 10.1371/journal.pone.0246125 (PMC7895410; doi:10.1371/journal.pone.0246125)
Supplement: S1 Table — Twenty-seven patients with cryptogenic allergy-like symptoms were examined rapid test with serum, determination of causative medicine in HiSAT or LTT. (PDF) [file pone.0246125.s005.pdf]

S1 Table. Details of patient and candidate medicine examied

Patient No.	Symptom	Candidate medicine	Company	Cmax&	Test conc.
1#	Other				
2#	Other				
3*,#	Eosinophilia	Mosapride	Sumitomo Dainippon Pharma Co., Ltd. (Osaka, Japan)	31 ng/mL	15 ng/mL
		Sulpiride	Astellas Pharma Inc. (Tokyo, Japan)	158 ng/mL	75 ng/mL
		Rabeprazole	Eisai Co., Ltd. (Tokyo, Japan)	406 ng/mL	200 ng/mL
4#	Other				
5#	Organopathy				
6#	Other				
7*	Eosinophilia	Paroxetine	GlaxoSmithKline K.K. (Tokyo, Japan)	6.48 ng/mL	5 ng/mL
8#	Organopathy				
9#	Rash				
10*,#,¶	Internal pneumonia	Kakkonto	Tsumura & Co. (Tokyo, Japan)	2500 mg/dose	100 μg/mL
11#	Organopathy				
12*	Rash	Phenitoin	Sumitomo Dainippon Pharma Co., Ltd. (Osaka, Japan)	2 μg/mL	5 μg/mL
		Phenobarbital	Fujinaga Pharm Co., Ltd. (Tokyo, Japan)	22.4 μg/mL	12.5 μg/mL
		Clonazepam	Chugai Pharmaceutical Co., Ltd. (Tokyo, Japan)	102 ng/mL	15 ng/mL
		Diazepam	Takeda Pharmaceutical Co., Ltd. (Tokyo, Japan)	1 μg/mL	50/500 ng/mL
		Midazolam	Sandoz K.K. (Tokyo, Japan)	102 ng/mL	50 ng/mL
13*,#	Organopathy	Clopidogrel	Sanofi K.K. (Tokyo, Japan)	3.94 μg/mL	2 μg/mL
		Aspirin	Bayer Yakuhin, Ltd. (Osaka, Japan)	0.45 μg/mL	0.25 μg/mL
14*,#	Organopathy	Tosufloxacin	Taisho Pharma Co., Ltd. (Tokyo, Japan)	1.06 μg/mL	0.5 μg/mL
15*,#	Other	Oxaliplatin	Yakult Honsha Co.,Ltd. (Tokyo, Japan)	1.6 μg/mL	0.4 μg/mL§
		Capecitabine	Chugai Pharmaceutical Co., Ltd. (Tokyo, Japan)	4.85 μg/mL	1.25 μg/mL§
16*,#	Organopathy	Hinyousen	Herbal medicine gifted from patient	unknown	0.006 - 60 mg/mL
		Bukuryo	Herbal medicine gifted from patient	unknown	2 mg/mL
		Mokutsu	Herbal medicine gifted from patient	unkown	2 mg/mL
		Byakujutsu	Herbal medicine gifted from patient	unknown	2 mg/mL
		Senkotsu	Herbal medicine gifted from patient	unknown	2 mg/mL
17*,#,¶	Eosinophilia	Levetiracetam	Otsuka Pharmaceutical Co., Ltd. (Tokyo, Japan)	16.4 μg/mL	10 μg/mL
		Lamotrigine	GlaxoSmithKline K.K. (Tokyo, Japan)	3.1 μg/mL	1 μg/mL
		Phenytoin	Sumitomo Dainippon Pharma Co., Ltd. (Osaka, Japan)	2 μg/mL	5 μg/mL
		Tazobactam/Piperacillin	Taisho Pharma Co., Ltd. (Tokyo, Japan)	36.3/286 μg/mL	30/120 μg/mL
18*,#	Rash	Cefazolin	Otsuka Pharmaceutical Co., Ltd. (Tokyo, Japan)	134 μg/mL	75 μg/mL
		Cefazolin	Astellas Pharma Inc. (Tokyo, Japan)	134 μg/mL	75 μg/mL
		Dimethylpolysiloxane	Kissei Pharmaceutical Co., Ltd. (Nagoya, Japan)	50 g/dose	1 μg/mL
19*,#,¶	Rash	Itraconazole	Janssen Pharmaceutical K.K. (Tokyo, Japan)	3201 ng/mL	100, 1000 ng/mL
		Itraconazole	Kaken Pharmaceutical Co., Ltd. (Tokyo, Japan)	215.6 ng/mL	100, 1000 ng/mL
20*,#,¶	Rash	Cefepime	Bristol-Myers Squibb. (Tokyo, Japan)	83.2 μg/mL	50μg/mL
		Tazobactam/Piperacillin	Taisho Pharma Co., Ltd. (Tokyo, Japan)	49.7/436 μg/mL	37.5/150 μg/mL
21*,#,¶	Rash	Phenytoin	Sumitomo Dainippon Pharma Co., Ltd. (Osaka, Japan)	10 μg/mL	5 μg/mL
		Thiopental	Mitsubishi Tanabe Pharma Corporation. (Osaka, Japan)	80 μg/mL	50 μg/mL
		Sodium valproate	Kyowa Kirin Co., Ltd. (Tokyo, Japan)	55 μg/mL	50 μg/mL
22*,#,¶	Organopathy	Famotidine	Nippon Chemiphar Co., Ltd. (Tokyo, Japan)	40.7 ng/mL	20 ng/mL
		Loxoprofen	Choseido Pharmaceutical Co.,Ltd. (Tokushima, Japan)	5 μg/mL	2.5 μg/mL
		Clarithromycin	Sandoz K.K. (Tokyo, Japan)	469.8 ng/mL	250 ng/mL
23*,#,¶	Organopathy	Rosuvastatin	AstraZeneca K.K. (Osaka, Japan)	3.56 ng/mL	1.5 ng/mL
		Ezetimibe	MSD K.K. (Tokyo, Japan)	6.03 ng/mL	5 ng/mL
		Amezinium	Sumitomo Dainippon Pharma Co., Ltd. (Osaka, Japan)	25.3 ng/mL	15 ng/mL
		Atorvastatin	Astellas Pharma Inc. (Tokyo, Japan)	2.64 ng/mL	1.5 ng/mL
24#	Rash	Levetiracetam	Otsuka Pharmaceutical Co., Ltd. (Tokyo, Japan)	16.4 μg/mL	10 μg/mL
		Topiramate	Kyowa Kirin Co., Ltd. (Tokyo, Japan)	0.84 μg/mL	0.5 μg/mL
		Carbamazepine	Novartis Pharma K.K. (Tokyo, Japan)	3.29 μg/mL	1.5 μg/mL
		Phenytoin	Sumitomo Dainippon Pharma Co., Ltd. (Osaka, Japan)	2 μg/mL	1 μg/mL
		Gabapentin	Pfizer Inc. (Tokyo, Japan)	2.48 μg/mL	1.5 μg/mL
		Lamotrigine	GlaxoSmithKline K.K. (Tokyo, Japan)	0.34 μg/mL	250 ng/mL
		Zonisamide	Sumitomo Dainippon Pharma Co., Ltd. (Osaka, Japan)	2.9 μg/mL	1.5 μg/mL
25*,#,¶	Organopathy	Ceftazidime	GlaxoSmithKline K.K. (Tokyo, Japan)	85.2 μg/mL	50 μg/mL
		Ampicillin/Sulbactam	Pfizer Inc. (Tokyo, Japan)	78.8/40.0 μg/mL	50/25 μg/mL
		Ceftriaxone	Chugai Pharmaceutical Co., Ltd. (Tokyo, Japan)	200 μg/mL	100μg/mL
26*,#,¶	Rash	Febuxostat	Teijin Pharma Ltd. (Tokyo, Japan)	496.2 ng/mL	250ng/mL
		Oxaliplatin	Yakult Honsha Co.,Ltd. (Tokyo, Japan)	1.6 μg/mL	0.4μg/mL§
27*,#,¶	Rash	Lamotrigine	GlaxoSmithKline K.K. (Tokyo, Japan)	0.72 μg/mL	0.25 μg/mL
		Levetiracetam	Otsuka Pharmaceutical Co., Ltd. (Tokyo, Japan)	16.4 μg/mL	10 μg/mL
		Phenytoin	Sumitomo Dainippon Pharma Co., Ltd. (Osaka, Japan)	2 μg/mL	1 μg/mL

#, Rapid test; *, Determination of causative medicine; ¶, LTT

&, Cmax was written on medical insert package or a dose in herbal medicine

§, Anticancer drug was used 1/4 of Cmax
