# Supplementary material for: Novel method to analyze cell kinetics for the rapid diagnosis and determination of the causative agent in allergy
Source: PLoS One. 2021 Feb 19;16(2):e0246125. doi: 10.1371/journal.pone.0246125 (PMC7895410; doi:10.1371/journal.pone.0246125)
Supplement: S1 Fig — Typical blot pattern at 3 of 10 concentrations of cytokine standard in flow cytometry with the Cytometric Bead Array (CBA) assay kit (A), typecal standard curve (B) and the r2 value that was fitted and was calculated using CBA software (C). The standard curves are well fitted, and the r2 values are greater than 0.998 under our experimental condition. (PDF) [file pone.0246125.s001.pdf]

A

B

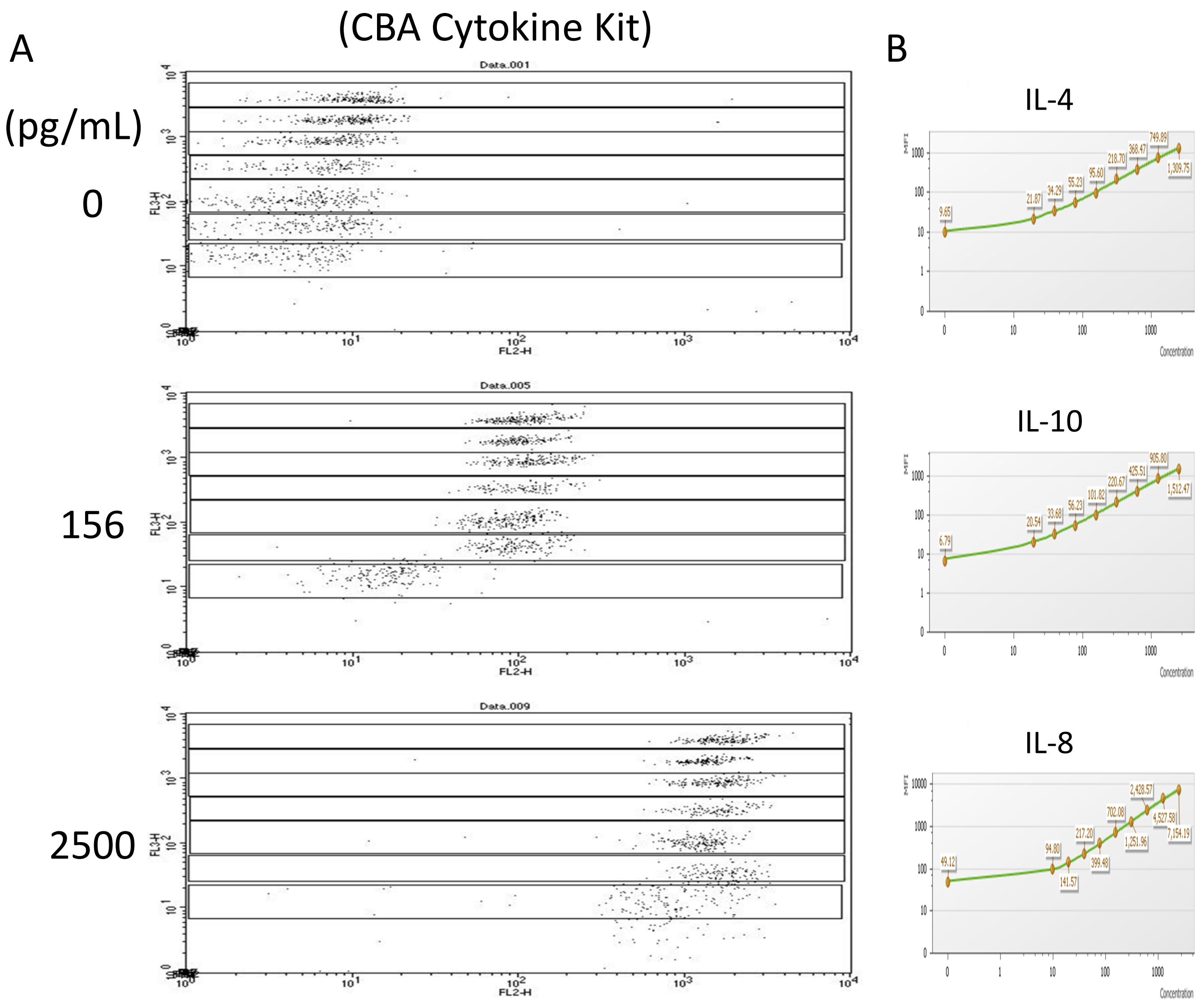

C

	r^2		r^2		r^2
IL-2	99.86	IFN-γ	99.98	RANTES	99.97
IL-4	99.91	TNF-α	99.86	MIG	99.94
IL-6	99.87	IL-17A	99.79	MCP-1	99.82
IL-10	99.94	IL-8	99.97	IP-10	99.84

(↑, Top) Supplementary Fig. S1. Shibaguchi H. et al.
